# The impact of COVID-19 on cultural industries: An empirical research based on stock market returns

**DOI:** 10.3389/fpubh.2022.806045

**Published:** 2022-09-16

**Authors:** Rong Zhang, Hao Ji, Yu Pang, Lingling Suo

**Affiliations:** ^1^Department of Finance and Accounting, Business College, Beijing Union University, Beijing, China; ^2^Department of Finance and Accounting, Management College, Beijing Union University, Beijing, China

**Keywords:** COVID-19, cultural industry, stock market, stock market return, fixed effects model

## Abstract

The COVID-19 virus has challenged the development of the cultural industries seriously, so far, however few studies have used empirical methods to analyze the impact of the pandemic on the overall cultural industries. Based on the panel data of listed companies, this paper explores the impact of COVID-19 on cultural industries from the perspective of stock market returns. The empirical results show that the pandemic has a significant negative impact on the stock market returns of cultural industries, but the degrees of impact on various creative sub-sectors are significantly different. The findings also indicate that digitalization can effectively reduce the negative impact of COVID-19 on cultural companies, and the epidemic has bigger negative impacts on small and newly-established cultural companies. Moreover, we find that the stock market returns of cultural industries have an inverted U-shaped relationship with the daily growth in total confirmed cases and in total cases of death caused by COVID-19, indicating that the negative marginal impact of COVID-19 on the cultural industries increases firstly and then gradually decreases. Finally, implications for companies and governments are presented respectively based on the findings.

## Introduction

The Black Swan Event of 2020—the outbreak of Covid-19, had a huge shock on the cultural industries (1–5). Taking China as an example, COVID-19 began to spread in China at the beginning of 2020. By the first quarter of 2020, the operating income of cultural, entertainment and leisure services fell by 59.1% compared with the same period of the previous year. Among them, entertainment services fell by 62.2%; cultural communication channels fell by 31.6%, and radio, film, television, and art performances, which represents offline consumption, fell by 78.5 and 46.2%, respectively^1^.

The stock market is a monitor that reflects the development of industries and enterprises. As the epidemic worsened, market sentiment spiraled out of control. On February 3, China's comprehensive daily market rate of return fell to the bottom, and the stock market fluctuated violently. The enterprises and whole industry suffered heavy losses. Judging from the performance of the cultural industries, on the first trading day after the Chinese Spring Festival, the cultural tourism sector of shares plunged 9.86% at the opening, and the cultural leisure and entertainment sector also fell by more than 9%.

Based on the sensitivity of the stock market to public emergencies, scholars have studied the stock market reaction of different industries, such as the accommodation industry (6), pharmaceutical industry (7), etc. However, there are few literatures about the impact of public emergencies on the cultural industries empirically.

The conclusions of the paper are similar to those of (8). Al-Awadhi's paper found that both daily growth in confirmed cases of COVID-19 in China and the growth rate of deaths are linearly negatively correlated with stock returns. However, there are differences between this paper and Al-Awadhi's paper. First, this paper focused on the impact of the epidemic on the cultural industries, rather than the overall industries. Second, the paper expanded the research conclusions of (8) and found that the heterogeneity of cultural companies, including industry type, digitalization level, company scale, and time to market, all affect its market response to the epidemic. Third, this paper also found that the stock market returns of cultural industries had an inverted U-shaped relationship with the severity of COVID-19, indicating that COVID-19 had an impact that strengthening first and weakening later on the stock market returns of the cultural industries.

The paper enriches the research about the economic consequences of infectious public health events. It focused on the impact of COVID-19 on the cultural industries and attempted to clarify the company heterogeneity in it. These are of great significance for government to response to public emergencies and to formulate targeted industry policies of cultural industries. It is also important for cultural enterprises to take emergency measures and for investors' awareness of the connection between public emergencies and stock market returns.

The rest paper is structured as follows: the second part is literature review and research hypothesis; the third part is sample selection and research design; the fourth and fifth parts are empirical analysis; and the final part is the conclusions and suggestions.

## Literature review and research hypothesis

### Research on the industry impact of infectious public health events

The uncertainty of infectious public health events may lead to negative psychological reactions like psychological imbalance, loss of control and other psychological reactions (9). The types of negative psychological reactions mainly include fear, anxiety, stress, and frustration. If most investors in the stock market are in this kind of psychology, they will be unwilling to invest, which will have a negative impact (10) and excessive market reaction on the stock market (11). For example, Ichev and Marine (12) found that the Ebola outbreak resulted in negative excess returns in the US stock market, and companies that had operations in the outbreak area would be more affected. Chen et al. (6) found that under the influence of SARS, the stock prices of listed accommodation companies in Taiwan Province of China generally fell during May 2003, with the largest drop of 29%. Similarly, infectious public health incidents also have a serious negative impact on the tourism industry (13, 14).

The impact of serious public emergencies on the development of the industry is also one of the important objects of this kind of research. Based on the impact of public emergencies on the development of the industry, existing research industries include commerce, tourism (15), hotel (16), etc. In terms of COVID-19, scholars have conducted research on the impact on the tourism (16), hotel and accommodation (17), dairy (18), energy industry (19) and some other industries. However, there are relatively few studies on the impact of COVID-19 on the cultural industries (2, 20–23), and they are mainly researches on a certain creative sub-industry, while the impact on the overall cultural industries is very small.

In terms of research methods, the existing research on the impact of covid-19 on cultural industries mainly uses case studies (24, 25), review of the literature (20) or government program analysis (26), but empirical methods are rarely used.

Therefore, this paper selected the panel data of listed companies in the cultural industries in China's stock markets. Based on stock market returns, fixed-effect model was adopted, empirically analyzing the impact of COVID-19 on the cultural industries. We also made suggestions for the government and creative enterprises to respond to public emergencies.

### Definition and classification of cultural industries

In the past few decades, the cultural industry has achieved tremendous development (27). Cultural industry is the main activity of producing and providing spiritual products to meet people's cultural needs as the goal, and refers to the creation and sale of cultural meaning itself. UNCTAD also provides a standard definition (28), which defines cultural industries as “any activities producing symbolic products with a heavy reliance on intellectual property and for as wide a market as possible” (29). Under the definition of UNCTAD, cultural industries include both traditional cultural industries such as publishing, broadcasting, television, film, performing arts, heritage and handicrafts, and cultural industry service industries such as advertising, architecture, design and photography (29–32).

### The impact of COVID-19 on cultural industries

At the beginning of 2020, COVID-19 pandemic began to spread in China. On January 23, 2020, Wuhan, where COVID-19 first be found in China, began to be locked down. Then various provinces and cities across China also imposed restrictions on the movement of people. The cultural industries have been hit hard by the sudden outbreak of COVID-19. Almost all offline entertainment activities, such as tourism, sports events, conventions, theaters and other gathering activities and places have been canceled and banned. Since the end of February, the epidemic has spread across the world, affecting as many as 200 countries and external environment on which the restoration of the cultural industry depends continued to deteriorate. COVID-19 has caused great damage to the cultural industries (2, 21, 23).

The cancellation of cultural events, exhibitions, concerts, performances and festivals caused by the epidemic, as well as restrictions on residents' activities and business activities in many countries, have had a negative impact on the cultural industry (3, 4, 23). The operations of many cultural companies have been disrupted, most of which were temporarily closed due to the epidemic. After resumption of work, they faced with a series of problems such as cash flow rupture, shortage of employees, suspension of production by upstream and downstream enterprises, and the unrecovery of consumer market. For example, XinChao Media, a well-known unicorn company, is the second largest cultural media company in China that focuses on community elevator advertising. Facing this sudden epidemic, it has carried out large-scale layoffs and salary cuts under huge pressure. The epidemic has caused many projects that have been invested by creative enterprises to a sudden halt and the costs could not be recovered in the short term. Banks and other financial institutions became more cautious while facing financing needs, leading to the collapse of corporate cash flow and difficulty in operating.

Furthermore, cultural companies generally have high requirements for the working environment and high mobility of personnel. In order to reduce operating costs, many companies often choose to rent sites, which made them fall into the awkward situation of suspension of production and work but still need to pay rent. Statistics show that more than 80% cultural companies' liquidity in the accounts can only maintain for 3 months. Cultural companies face great operating pressure, and some companies fail to maintain their operations and go bankrupt.

The stock market is the “barometer” of the development of the industry. After the outbreak of COVID-19, the major global stock markets have plummeted, setting a record for the decline in recent years. In terms of China's cultural industry, on the first trading day after the Chinese Spring Festival, the cultural and tourism sector of A-shares dropped 9.86% at the opening, and the cultural, Leisure and entertainment sector also fell by more than 9%. The epidemic has caused insufficient effective demand for the cultural industry and poor corporate operating efficiency. As a result, the development of the cultural industry faced huge challenges. The reflection in the stock market was investor pessimism, stock market volatility and decline in stock market returns. Therefore, we propose the following hypothesis:

H1: The stock market returns on the cultural industries are negatively correlated with the severity of COVID-19.

### The impact of COVID-19 on different creative sub-industries

Existing studies have shown that infectious public health events have different impacts on the performance of listed companies in different industries, leading to different stock returns (8). Ichev and Marine (12) found that during the Ebola outbreak, the stock returns of the US medical device manufacturing, pharmaceutical, biotech, and food manufacturing industries were all significantly positive, while those of other industries were all negative. Chen et al. (33) found that SARS caused the market value of listed companies in the tourism, retail, and railway transportation industries in Taiwan Province to plunge, while it had a positive impact on the biotechnology industry.

Belitski (20) divides the different situations of creative sub-industries under COVID-19 into four categories. The first type of creative sub-industries is low digital capabilities and low ability to adapt, such as the music industry, theaters, etc. These companies are facing serious crises or even bankruptcy under the impact of COVID-19. The second type of creative enterprises belong to the types of high digital capabilities and low ability to adapt, such as social media, publishing, and journalism. They can still barely maintain and earn a small amount of income during the epidemic. And industries such as IT and software belong to high digital capabilities and high ability to adapt, and they can continue to grow during the epidemic period. The last category is low digital capabilities and high ability to adapt, such as museums, libraries, exhibition industries, which may suspend operations and cope with COVID-19 through government funding and cost reduction until they are allowed to reopen (20).

Due to differences in the service characteristics, venue requirements, and customer demand flexibility of cultural enterprises, the impact of the epidemic on the market returns of different types of creative sub-sectors is obviously different. Therefore, we propose the following hypothesis:

H2: There are significant differences in the impact of the epidemic on the stock market returns of different creative sub-sectors.

### The moderating effect of digitalization on the impact of the epidemic

Under the wave of new technologies, the digital cultural industry has been showing a trend of vigorous development in recent years. The epidemic has also caused some museums to offer online exhibitions, and musicians provide online concerts or record their performances (24, 34, 35). Digital tools and digital services are considered a safe and effective way for cultural companies to maintain normal operations and growth during the epidemic (36).

During the epidemic, traditional offline cultural companies suffered severe negative impacts, while emerging digital cultural companies have highlighted their advantages due to the characteristics of online consumption and gained a larger market share because hundreds of millions of people are isolated from home and work remotely. Correspondingly, the stocks of cultural companies with higher levels of digitalization are increasingly sought after by the capital market. For example, the share price of China Reading Group, the leading digital reading company listed on the Hong Kong Stock Exchange, bucked the trend and rose on February 3.

Digital technology also has a significant impact on some traditional creative enterprises. Take Songcheng Performing Arts, a listed performing arts company in China, as an example. In 2018, the company's annual report pointed out that the company will actively participate in online entertainment through cooperation with Internet companies, and strive to create an ecosystem of offline performing arts and online entertainment. On February 3, when China's cultural, leisure and entertainment sector fell by more than 9%, the stock price of Songcheng Performing Arts, which deployed online entertainment business in advance, fell only 4.60% on the trading day. The increase in digitalization has offset the impact of the epidemic on the cultural industries to a certain extent. Therefore, we propose the following hypothesis:

H3: The digitalization of creative enterprises can effectively reduce the negative impact of COVID-19 on the cultural industries.

## Sample selection and research design

### Sample selection and data source

At the beginning of 2020, COVID-19 began to spread in China. On January 23, 2020, Wuhan began to “close,” urban buses and subways were temporarily suspended, and passages such as stations and airports were temporarily closed. Subsequently, various provinces and cities in China also imposed restrictions on the movement of people.

Since the Chinese Health Commission began to release data related to COVID-19 on January 11, 2020, this paper regards it as the starting point of this research. Therefore, in order to observe the continuing impact of the epidemic on the cultural industries, the research period of this paper is from January 11, 2020 to July 15, 2020.

This paper selects cultural companies listed on the Shanghai and Shenzhen Stock Exchange in China as the initial sample. In terms of the definition of creative enterprises, the UNCTAD (29) definition of cultural industries was adopted and combined with the 2012 edition of the China Securities Regulatory Commission's industry classification. Therefore, the sub-sectors of the cultural industry in this paper mainly include (1) news and publishing industry, (2) film, TV, and broadcasting related industries (3) culture and art industry (4) sports industry.

After excluding cultural companies with special treatment or missing data, there are a total of 54 Chinese listed cultural companies with 4,735 valid sample. The data comes from the CSMAR database and the annual reports of these cultural companies. All the continuous variables are winsorized at 99% percentiles.

### Model specification and variable definition

The paper did not use the traditional event method to conduct research as COVID-19 pandemic is a continuous event. Hsiao (37) believed that panel data regression can reduce prediction bias and multicollinearity, control individual heterogeneity, and identify the dynamic relationship between the explained variable and the explanatory variable. Therefore, after controlling the individual characteristics of the company, we used the panel data fixed-effect model to investigate the impact of COVID-19 on stock returns of cultural industry. Drawing on the research of Al-Awadhi (8) and Ichev and Marine (12), the paper sets up models (1) and (2) to test the hypotheses. The variable definitions are shown in Table 1.


(1) $$\begin{array}{l} \begin{array}{c} DR_{i,t}=\alpha_{0}+\beta_{1}DGCC_{t-1}+\beta_{3}MTB_{t-1} \end{array}\\ \;\begin{array}{c} +\beta_{4}LnMCAP_{t-1}+\varepsilon \end{array} \end{array}$$

(2) $$\begin{array}{l} \begin{array}{c} DR_{i,t}=\alpha_{0}+\beta_{1}DGDC_{t-1}+\beta_{3}MTB_{t-1} \end{array}\\ \;\begin{array}{c} +\beta_{4}LnMCAP_{t-1}+\varepsilon \end{array} \end{array}$$

This paper uses the daily growth in COVID-19 confirmed cases and the daily growth in death cases from COVID-19 to measure the severity of the epidemic. The stock return of cultural companies is used as an explained variable to measure the stock market return of cultural industries. In order to control the heterogeneous selection of individual companies, daily market-to-book ratio and daily market capitalization are used as control variables. The variable description is shown in Table 1.

**Table 1:** Variable definition and description.

**Variable**	**Abbr**.	**Definition**
Dependent variable	DR_i, t_	Daily stock return of company *i* on day *t*
Independent variable	DGCC_t−1_	Daily growth in confirmed cases, that is, the number of newly confirmed cases on day t-1 divided by the cumulative number of confirmed cases on the previous day
	DGDC_t−1_	Daily growth in death cases from COVID-19, that is, the number of new deaths on day t-1 divided by the cumulative number of deaths in the previous day
Control variable	MTB_i, t−1_	Daily market-to-book ratio/1,000 of company *i* on t-1 day
	lnMCAP_i, t−1_	the natural logarithm of daily market capitalization of company *i* on day t-1

## Empirical results analysis

### Descriptive statistics

As shown in the statistical results in Table 2, the average DR is 0.003; the minimum is −0.0772; the maximum is 0.1, suggesting that the stock returns of listed companies are not consistent in the positive and negative directions during COVID-19 pandemic. There may be two reasons for this phenomenon. First, companies with different industry classifications in the cultural industry are affected differently by the epidemic. For example, due to restrictions on public activities during the epidemic, the film and performance industry were more affected. With its performance bleak, its stock prices inevitably fell. Second, with the effective intervention of the government, the development of the epidemic, information disclosure and economic policies have gradually become clear. The uncertainty caused by the epidemic continued to decrease, and the mentality of investors became more rational. Therefore, stock prices that have fallen excessively at the beginning of the epidemic got retaliatory increases.

**Table 2:** Descriptive statistics.

**Variable**	***N***	**Mean**	**Sd**.	**Min**.	**Max**.
DR	4,735	0.003	0.030	−0.077	0.100
DGCC	4,735	0.022	0.073	0.000	0.454
DGDC	4,735	0.032	0.112	0.000	0.889
LnMCAP	4,735	22.320	0.986	20.370	24.940
MTB	4,735	0.003	0.003	0.001	0.030

DR, daily stock return; DGCC, daily growth in confirmed cases of COVID-19; DGDC, daily growth in death cases from COVID-19; LMCAP, natural logarithm of daily firm market capitalization/1,000; MTB, daily market-to-book ratio.

### Multiple regression analysis

*P*-value of the *F*-test is 0.000, suggesting the mixed estimation model is rejected at the significance level of 1%; *P*-value of the Hausman test is also 0.000, indicating that the random effect model is rejected, thereby we adopted a fixed effect model to regress.

#### COVID-19 and stock returns

Column (1), (2), and (3) of Table 3 list the regression results of the confirmed growth rate on stock returns using the panel fixed effects model. Among them, column (1) is the regression result without adding control variables, and column (2) and (3) are the regression results after adding control variables. The regression results show that the regression co-efficients of daily growth in confirmed cases (DGCC) are all significantly negative, and they all pass the significance test at the 1% level, and the null hypothesis was accepted.

**Table 3:** Panel regression.

	**(1)**	**(2)**	**(3)**	**(4)**	**(5)**	**(6)**
DGCC	−0.022*** (−2.67)	−0.021** (−2.57)	−0.021** (−2.57)			
DGDC				−0.031*** (−6.89)	−0.030*** (−6.72)	−0.030*** (−6.75)
LnMCAP		−0.022*** (−5.45)	−0.030*** (−6.19)		−0.021*** (−5.27)	−0.029*** (−6.09)
MTB			3.412*** (3.02)			3.469*** (3.09)
_cons	0.001** (2.44)	0.481*** (5.46)	0.663*** (6.22)	0.002*** (3.49)	0.464*** (5.29)	0.649*** (6.12)
*N*	3,278	3,278	3,278	3,278	3,278	3,278

This table reports the co-efficients of the panel regressions results for cultural companies listed on the Shanghai and Shenzhen stock Exchange from January 11, 2020 to July 15, 2020. Column (1), (2), and (3) reports the co-efficients of the panel regressions for daily growth rate in confirmed cases (DGCC). Column (4), (5), and (6) reports the co-efficients of the panel regressions for daily growth rate in death cases (DGDC). The dependent variable is DR_i, t_, which is the daily stock return of company i on day t. LnMCAP, natural logarithm of daily firm market capitalization; MTB, daily market-to-book ratio divided by 1,000. T statistics are in parentheses; **, *** denote statistical significance at the 10%, 5%, and 1% levels, respectively.

Column (4), (5), and (6) of Table 3 report the regression result of another indicator of the severity of epidemic, namely the growth rate of death and stock returns. The co-efficients of the death growth rate (DGDC) are also significantly <0 at the 1% level, indicating that the death growth rate and stock returns are also negatively correlated. Therefore, the hypothesis H1 that the returns on the cultural industry stock market are negatively correlated with the severity of the epidemic has been verified.

#### Sector analysis

According to the 2012 edition of the China Securities Regulatory Commission's industry classification, there are four types of cultural industry subordinates, namely (1) press and publication industry, (2) Film, TV, and broadcasting related industries (listed companies are mainly in the film industry) (3) culture and art industry (4) sports industry. To explore whether there are differences in stock market returns for different types of cultural industries affected by COVID-19, we took these four types of listed companies as the research objects, and substitute their relevant data into the fixed effects model. The results are shown in Tables 4, 5.

**Table 4:** Panel regression with specific sectors dummy variable.

	**Press and publication industry**	**Film industry**	**Culture and art industry**	**Sports industry**
**Panel A: Daily growth in confirmed cases**				
DGCC	−0.003 (−0.25)	−0.025* (−1.87)	−0.030 (−1.25)	−0.067 (−1.46)
LnMCAP	−0.004 (−0.53)	−0.071*** (−6.91)	−0.045** (−2.11)	−0.002 (−0.12)
MTB	−9.457** (−2.26)	7.275*** (5.02)	6.556 (1.55)	−18.892 (−1.49)
_cons	0.107 (0.66)	1.548*** (6.91)	1.002** (2.13)	0.101 (0.34)
*N*	1,464	1,334	419	61

This table reports the co-efficients of the panel regressions results for cultural companies listed on the Shanghai and Shenzhen stock Exchange from January 11, 2020 to July 15, 2020, considering specific sectors. The dependent variable is DR_i, t_, which is the daily stock return of company i on day t. Press and publication, film industry, culture and art industry, sports industry. are sector dummy variables that take the value one if the stock is listed in that respective sector, and zero otherwise. LnMCAP, natural logarithm of daily firm market capitalization; MTB, daily market-to-book ratio divided by 1,000. T statistics are in parentheses. *, **, *** denote statistical significance at the 10%, 5%, and 1% levels, respectively.

**Table 5:** Panel regression with specific sectors dummy variable.

	**Press and publication industry**	**Film industry**	**Culture and art industry**	**Sports industry**
**Panel B: Daily growth in death cases from COVID-19**				
DGDC	−0.017*** (−2.66)	−0.037*** (−5.10)	−0.030** (−2.34)	−0.039 (−1.59)
LnMCAP	−0.005 (−0.73)	−0.065*** (−6.42)	−0.043** (−2.02)	−0.001 (−0.09)
MTB	−8.116* (−1.94)	6.926*** (4.82)	6.137 (1.46)	−16.400 (−1.28)
_cons	0.138 (0.86)	1.432*** (6.41)	0.955** (2.04)	0.083 (0.28)
*N*	1,464	1,334	419	61

This table reports the co-efficients of the panel regressions results for cultural companies listed on the Shanghai and Shenzhen stock Exchange from January 11, 2020 to July 15, 2020, considering specific sectors. The dependent variable is DR_i, t_, which is the daily stock return of company i on day t. Press and publication, film industry, culture and art industry, sports industry are sector dummy variables that take the value one if the stock is listed in that respective sector, and zero otherwise. LnMCAP, natural logarithm of daily firm market capitalization; MTB. daily market-to-book ratio divided by 1,000. T statistics are in parentheses. *, **, *** denote statistical significance at the 10%, 5%, and 1% levels, respectively.

The regression results in Table 4 show that in the film industry group, the regression co-efficient of daily growth in confirmed cases (DGCC) is −0.025, which is significant at the 10% level; in other groups, the regression co-efficient of DGCC failed the significance test. We further tested the regression results of death growth rate (DGDC) and stock returns in different types of cultural industry groups. As shown in Table 5, the regression co-efficients of the three creative sub-sectors (1) the press and publication industry; (2) film production industry; (3) the culture and art industry, are all negative and have passed the significance test. However, the film industry still has the highest level of significance and the largest absolute value of the co-efficient. This shows that COVID-19 mortality rate still has the most significant negative impact on the film industry.

The greater impact of the epidemic on movies and related industries stems from industries such as movies and performing arts, and the most negative impact was suffered during the epidemic. Taking China as an example, movie box office data show that the box office of the Spring Festival in 2019 was 5.9 billion, accounting for nearly 10% of the annual box office. In the 2020 Spring Festival season, all the seven New Year films of 2020, known as the strongest in China's history, were forced to be taken off the shelves. Various agencies have predicted that the box office of the 2020 Chinese New Year would exceed 10 billion, but it was only 23.57 million finally. Huge losses have brought financial difficulties to film companies, led a crisis of survival, and resulted in a sharp decline in film production capacity in 2020. On the other hand, various theater crews were also affected by the prevention and control of the epidemic, and could not produce film and television dramas in the first quarter of 2020. The original production plan of the work has been shelved, making the prospects of film and television recording industries serious.

#### The moderating effect of digitalization

We used manual sorting and automatic word segmentation with Python algorithm to determine the basic ways of different companies in the annual report to express digitization-related information. The final selected keywords are shown in Table 6. On the basis of obtaining specific keywords for digital transformation, utilizing big data crawler function of Python, the text of the annual report of listed companies in the cultural and cultural industries in 2019 is captured. We matched it with the keywords in Table 6, counted the number of occurrences of each keyword in the annual report, and then totaled up them to obtain the total indicators of the company's digital level. According to this digital indicator, we sorted from high to low, and then divided cultural companies into two groups: high-digital level and low-digital level.

**Table 6:** Keywords related to enterprise digitalization in the cultural industry.

Mobile internet, mobile internet, E-commerce, smart cultural tourism, smart marketing, digital marketing, unmanned retail, Internet Finance, digital finance, Fintech big data, data mining, text mining, data visualization, heterogeneous data, augmented reality, mixed reality, virtual reality, online, ecological collaboration, online retail, internet+, business smart, digital city, digital creativity, digital divide, digital business, digital, digital technology, knowledge management, smart office, smart terminal, natural language processing, internet, relational database, machine learning, deep learning, data empowerment, data visualization, data cleaning, network security, cloud storage, cloud computing, cloud networking, cloud platform, platform economy, internet of things, etc.

Table 7 is the regression results of the impact of different digitalization levels on the relationship between COVID-19 and the stock market return. The first and third columns and the fourth and fifth columns are the groups with low and high digitization degree, respectively. When the company is at a low degree of digitalization, the regression co-efficient of daily growth in confirmed cases (DGCC) is −0.0348, and it is significant at 1% level, When the company is at a high degree of digitalization, the regression co-efficient of daily growth in confirmed cases (DGCC) reduced greatly to −0.007, and the significance test is not passed. This statistical result shows that a higher degree of digitization can effectively reduce the negative impact of COVID-19 on stock market returns.

**Table 7:** The impact of firm digitalization on market response to COVID-19.

**Digitalization degree**	**Low**	**High**	**Low**	**High**
DGCC	−0.0348*** (−3.25)	−0.0070 (−0.55)		
DGDC			−0.0335*** (−3.16)	−0.0069 (−0.54)
LnMCAP			−0.0002 (−0.21)	−0.0002 (−0.25)
MTB			0.1011 (0.34)	0.4542* (1.68)
_cons	−0.0027*** (−3.57)	0.0055*** (6.48)	0.0007 (0.04)	0.0085 (0.48)
*N*	1,598	1,598	1,598	1,598

This table reports the co-efficients of the panel regressions results for cultural companies listed on the Shanghai and Shenzhen stock Exchange from January 11, 2020 to July 15, 2020, considering digitalization. Column (1), (2) reports the co-efficients of the panel regressions for daily growth rate in confirmed cases (DGCC). Column (3), (4) reports the co-efficients of the panel regressions for daily growth rate in death cases (DGDC). The dependent variable is DR_i, t_, which is the daily stock return of company i on day t. The enterprise digitalization level indicators are calculated and sorted. Those in the top 50th percentile are classified as high-digitization level groups (columns 2 and 4), and those in the bottom 50th percentile are classified as low-digitization level groups (columns 1, 3). LnMCAP, natural logarithm of daily firm market capitalization; MTB, daily market-to-book ratio divided by 1,000. T statistics are in parentheses; *, *** denote statistical significance at the 10%, 5%, and 1% levels, respectively.

## Further analysis

### The impact of firm size and age in market response to COVID-19

The large scale of a company represents a complete corporate governance system, reasonable staffing, outstanding industry status and standardized information disclosure to a certain extent. Existing studies showed that the larger the company, the greater they respond to good news and the stronger the immunity to bad news Schwert (38). Brown and Cliff (39) found that if investor sentiment is optimistic, the company's market value will be significantly greater than its embedded value, and this effect is more significant in large-scale companies. Schwert (38) found that in the 1987 US stock market crash, the market value of large companies fell less, and recovered faster and more after crash. Ichev and Marine (12) also found that the Ebola epidemic has less impact on larger companies. After the outbreak of COVID-19, although all creative enterprises faced the same external environment, large-scale enterprises were less affected by the epidemic due to their strong operating capabilities. Therefore, we expect that the stock prices of larger cultural companies will be less affected by the epidemic.

Similar to the company size indicator, some scholars argue that the company's age has a positive linear relationship with the company's growth rate 1 (40). Carroll (41) proposed that as companies grow older, they have accumulated profound operating experience, have strong environmental adaptability, and the ability to maintain and update resources, so the company's viability improves. Similarly, Jensen et al. (42) also found that the productivity of enterprises increases with age. Therefore, we assume that the stock prices of cultural companies with a longer listing age will be less affected by the epidemic.

We divided companies into three equal groups according to the scale of their assets, and then compared the market response to the epidemic of the smallest group and the largest group.

As shown in Table 8, in the regression of market response and DGCC, small-scale companies' are significantly negative, while large-scale companies have insignificant co-efficients. In the regression of market response and DGDC, although the co-efficients of small-scale and large-scale companies are both significant, the absolute value and significance of the co-efficients of small-scale companies are higher than those of large-scale companies. The results suggest that large market small companies experience significantly more negative effect on returns than larger companies.

**Table 8:** The impact of firm size on market response to COVID-19.

	**Small-scale**	**Large-scale**	**Small-scale**	**Large-scale**
DGCC	−0.035** (−2.30)	0.009 (0.70)		
DGDC			−0.037*** (−4.44)	−0.019*** (−2.78)
LnMCAP	−0.017*** (−2.64)	−0.100*** (−7.50)		−0.091*** (−6.79)
MTB	3.017** (2.22)	12.271*** (4.10)		11.061*** (3.71)
_cons	0.361*** (2.62)	2.301*** (7.53)	0.003*** (2.64)	2.076*** (6.81)
*N*	1,143	1,037	1,143	1,037

This table reports the co-efficients of the panel regressions results for cultural companies listed on the Shanghai and Shenzhen stock Exchange from January 11, 2020 to July 15, 2020, considering firm size. Column (1), (2) reports the co-efficients of the panel regressions for daily growth rate in confirmed cases (DGCC). Column (3), (4) reports the co-efficients of the panel regressions for daily growth rate in death cases (DGDC). The dependent variable is DR_i, t_, which is the daily stock return of company i on day t. According to the size of assets, the cultural companies are sorted and divided into 3 groups equally, and the smallest group (columns 1, 3) and the largest group (columns 2, 4) are respectively used for regression. T statistics are in parentheses; **, *** denote statistical significance at the 10%, 5%, and 1% levels, respectively.

Similarly, we also divided the cultural companies into three equal groups according to their listing age, and then compared the market response to the epidemic of the shortest listing age group and the longest listing age group. As shown in Table 9, in the regression of market response and DGCC, the co-efficient for the short-listed age group is significantly negative, while the co-efficient for mature companies with a longer age is not significant. In the regression of the market response and DGDC, the absolute value and significance of the co-efficient of the shortest-term group are higher than those of large-scale enterprises. The results show that the epidemic has a significantly lower negative impact on the stock returns of mature cultural companies that have been listed for a long time than young companies.

**Table 9:** The impact of firm age on market response to the COVID-19.

	**Short listed period**	**Long listed period**	**Short listed period**	**Long listed period**
DGCC	−0.034*** (−2.62)	−0.001 (−0.04)		
DGDC			−0.035*** (−5.07)	−0.020** (−2.54)
LnMCAP	−0.013** (−2.17)	−0.055*** (−2.95)		−0.051*** (−2.74)
MTB	2.870** (2.40)	−3.883 (−0.42)		−3.685 (−0.40)
_cons	0.271** (2.15)	1.250*** (3.06)	0.003*** (3.33)	1.162*** (2.86)
*N*	1,330	1,037	1,330	1,037

This table reports the co-efficients of the panel regressions results for cultural companies listed on the Shanghai and Shenzhen stock Exchange from January 11, 2020 to July 15, 2020, considering firm age. Column (1), (2) reports the co-efficients of the panel regressions for daily growth rate in confirmed cases (DGCC). Column (3), (4) reports the co-efficients of the panel regressions for daily growth rate in death cases (DGDC). The dependent variable is DR_i, t_, which is the daily stock return of company i on day t. According to the listing age, the cultural companies are sorted and divided into three groups equally, and the shortest listing age group (columns 1, 3) and the longest listing age group (columns 2, 4) are respectively used for regression. T statistics are in parentheses; **, *** denote statistical significance at the 10%, 5%, and 1% levels, respectively.

### The changing trend of the impact of COVID-19 on the market response

According to statistics, death rate of COVID-19 in China is far lower than 9% of influenza pneumonia and 10% of SARS. However, at the beginning of the epidemic, due to ignorance of it, lack of clear information channels and uncertain future economic development expectations, stock investors were generally panic and pessimistic, resulting in stock market volatility. However, with the strong and effective intervention of the government, the development of the epidemic, information disclosure, and economic policies gradually became clear. Uncertainty continued to decrease, and investors became more rational. Therefore, even if the epidemic still exists, its marginal impact on the stock market will be reduced significantly. Therefore, we assume that the severity of the epidemic has an inverted U-shaped relationship with stock market returns. The paper expects that COVID-19 will have a marginal impact on the stock market returns of the cultural industry that will first increase and then weaken.

Column (1), (2), and (3) of Table 10 show the regression results of daily growth in confirmed cases and stock returns. Among them, the co-efficient of DGCC^2^ is significantly negative. Turning point 0.1315 is located within the data range, so the turning point and the observed value to the right can be covered. This means that there is a non-linear “U” relationship between daily growth in confirmed cases and stock returns. Before the turning point, stock returns will decline with the increase of the confirmed growth rate, after reaching the turning point, it will rise. Column (4), (5), and (6) of Table 10 show the regression results of death growth rate and stock return. Similar to the confirmed growth rate, the co-efficients of DGDC^2^ in the three regressions are all significantly negative. The turning point 0.2973 is within the range of the data, indicating that the death growth rate and stock return are also in an inverted U-shaped relationship. Therefore, in line with our expectations, the severity of the epidemic has an inverted U-shaped relationship with stock market returns. The impact of COVID-19 on the stock market returns of cultural industries first increases and then gradually weakens.

**Table 10:** Changing trend of the COVID-19's impact on the stock returns in cultural industry.

	**(1)**	**(2)**	**(3)**	**(4)**	**(5)**	**(6)**
DGCC	0.364*** (13.12)	0.360*** (13.04)	0.362*** (13.11)			
DGCC^2^	−1.386*** (−14.53)	−1.371*** (−14.42)	−1.376*** (−14.49)			
DGDC				0.085*** (5.89)	0.087*** (6.08)	0.088*** (6.15)
DGDC^2^				−0.145*** (−8.43)	−0.147*** (−8.58)	−0.148*** (−8.66)
LnMCAP		−0.020*** (−5.15)	−0.029*** (−6.15)		−0.021*** (−5.51)	−0.031*** (−6.41)
MTB			3.674*** (3.36)			3.667*** (3.30)
_cons	−0.000 (−0.63)	0.440*** (5.15)	0.636*** (6.15)	0.000 (0.52)	0.478*** (5.51)	0.673*** (6.42)
*N*	3,278	3,278	3,278	3,278	3,278	3,278

This table reports the co-efficients of the panel regressions results for cultural companies listed on the Shanghai and Shenzhen stock Exchange from January 11, 2020 to July 15, 2020, considering trends in the impact of the epidemic. Therefore, the square terms of DGCC and DGDC are added to the regression model. Column (1), (2), and (3) reports the co-efficients of the panel regressions for daily growth rate in confirmed cases (DGCC). Column (4), (5), and (6) reports the co-efficients of the panel regressions for daily growth rate in death cases (DGDC). The dependent variable is DR_i, t_, which is the daily stock return of company i on day t. T statistics are in parentheses; *** denote statistical significance at the 10%, 5%, and 1% levels, respectively.

## Conclusions and suggestions

### Research conclusions

Utilizing the fixed effects model of panel data, the paper studied the impact of COVID-19 pandemic on the return of cultural industry stocks. We also examined the impact of company heterogeneity on the market response to COVID-19. The study found that the return of the cultural industry stock market is negatively correlated with the severity of COVID-19. Second, different types of cultural industries are affected by the epidemic significantly differently, and the film industry, for example, has been significantly negatively affected. Third, digitization can reduce the negative impact of COVID-19 on the cultural industry greatly. Further research shows that the negative impact of the epidemic on the stock returns of small-scale and younger cultural companies is more serious. Our empirical results also show that COVID-19 has a marginal impact on the stock returns of the cultural industry that strengthened first and gradually weakened later.

### Implications for companies

The conclusion of this paper shows that the anti-risk ability of highly digitalized cultural companies is significantly higher than that of less digitalized companies. In fact, the COVID-19 pandemic has forced cultural companies to undergo extensive digital transformation (34, 43). Cultural companies, especially traditional cultural companies, should accelerate the pace of technological innovation. Companies should strengthen the application of 5G, VR, AR, big data, artificial intelligence, blockchain, and other technologies, change the industry's traditional production and operation methods that were single, decentralized, and less intensive in the past, and actively seize the digital consumer market. This is important for cultural companies to prepare for a new business model for the post-epidemic era and other possible public emergencies in the future. For example, the convention and exhibition industry has derived an “online exhibition” model, and the publishing industry has accelerated its deployment to “digital publishing” and “digital reading”(44).

Meantime, there are more and more cross-industry behaviors being quietly carried out (45). The emergence of “shared employees” and “shared scenes” has provided a new model for industry and enterprise collaboration. The application of media digital technology has a positive impact on social governance and omni-media integration. Short video, webcast, UGC and other digital communication methods also brought more cross-border integration to traditional industries such as catering, clothing, construction, and e-commerce market. It is foreseeable that the application of digital technology will blur the boundaries between different industries and further enhance liquidity. The digital cultural industry will penetrate more widely into all walks of life, achieving a deeper, more diversified, and more directional integration.

Creative enterprises also need to optimize their own structure to become stronger and bigger. Most cultural companies are small in scale and have a single business model, so their profits are usually relatively low. However, as the empirical results of this paper show, the negative impact of the epidemic on the market returns of smaller cultural companies is significantly higher than that of large-scale companies. With the increasing demand of residents for high-quality cultural and sports leisure services, the cultural industry should continue to strengthen the adjustment of its own scale and industrial structure while ushering in a broader market prospect. Take Disney as an example, its business includes the entire industry chain of film and television entertainment, television media, new media communications, Disneyland and consumer products. In 2018, its operating income reached 59.4 billion U.S. dollars and net profit was 12.6 billion U.S. dollars. After the outbreak of the epidemic, the cultural industry suffered a greater impact in the short term, especially cultural tourism and leisure service companies. On the bright side, this also provides opportunities for mergers and reorganizations of leading companies. Therefore, some high-quality cultural companies may expand their competitive advantages and market share through mergers or reorganizations, and stand out.

### Implications for government

The research shows that the impact of COVID-19 on different types of cultural industries is significantly different. For creative sub-sectors such as performing arts and film industry that have been greatly negatively affected, it is recommended that the government use loan interest discounts, tax reductions, rent reductions, financial support and other supporting policies to help them overcome difficulties in production and operation.

On the one hand, these companies should be encouraged to carry out more service innovation and to improve the level of digitalization, such as the development of network theaters, online performing arts and other digital business formats. On the other hand, according to the findings of Hylland (34), the recent rapid digitization of cultural enterprises was forced to occur due to the epidemic, rather than spontaneous changes in consumer behavior or more efficient cultural service tools, and many cultural providers and consumers hope to return to a normal, less digital situation. Therefore, once the epidemic is relieved, various offline cultural activities should be resumed and policies should be adopted to stimulate offline cultural consumption in order to alleviate the pressure on these severely negatively affected industries.

Our conclusion also show that the negative impact of the stock market on larger creative enterprises with longer years is significantly less than that of younger SMEs (small and medium-size enterprises). Small, medium and micro enterprises and young creative enterprises are extremely vulnerable to shocks in the face of public emergencies, and then face severe survival dilemmas. Therefore, SMEs or young creative enterprises should be given certain support policies, with preferential treatment in terms of bank loans, rent subsidies, tax reductions and exemptions. These policies will alleviate the pressure of survival for these small enterprises and establish a corporate ecological environment that resists the risks that may arise in the process of creative enterprises and their digital transformation.

In addition, some scholars suggest establishing a stronger support for cultural companies and their practitioners through third-party organizations such as professional associations (20). Due to economies of scale, this centralized third-party organization will provide creative SMEs with databases, artist relationships, and partner networks with lower management costs, and in short, can provide better overall support.

Finally, this paper shows that the impact of COVID-19 on the stock returns of the cultural industry first strengthened and then gradually weakened. This phenomenon is mainly due to the public's irrational and pessimistic judgments about the future situation in the early stage of the epidemic, caused by the suspension of business and production. With the effective intervention of the government, the epidemic, information disclosure and economic policies have gradually become clear. The uncertainty caused by the epidemic continued to decrease, and the mentality of investors became more rational. The impact of COVID-19 on the return of the cultural industry's stock market has gradually weakened. Therefore, the impact of infectious public health events on the economy is largely due to the public's uncertainty about the infectious disease itself and its economic consequences. To control these uncertainties, the infectious disease prevention and control system needs to be further strengthened. The authority of the National Center for Disease Control and Prevention should be increased in order to detect and disclose any new epidemics in a timely manner.

## Data availability statement

Publicly available datasets were analyzed in this study. This data can be found here: https://www.gtarsc.com/.

## Author contributions

HJ: conceptualization, methodology, software, formal analysis, resources, and writing—original draft preparation. RZ, YP, and HJ: validation. RZ, HJ, YP, and LS: data curation and writing—review and editing. All authors have read and agreed to the published version of the manuscript.

## Funding

This research was funded by Institute of Beijing Studies (BJXJD-KT2022-YB07).

## Conflict of interest

The authors declare that the research was conducted in the absence of any commercial or financial relationships that could be construed as a potential conflict of interest.

## Publisher's note

All claims expressed in this article are solely those of the authors and do not necessarily represent those of their affiliated organizations, or those of the publisher, the editors and the reviewers. Any product that may be evaluated in this article, or claim that may be made by its manufacturer, is not guaranteed or endorsed by the publisher.

## Footnotes

^1^According to data released by the National Bureau of Statistics of China.
